# Dielectric analysis and electrical conduction mechanism of La_1−*x*_Bi_*x*_FeO_3_ ceramics

**DOI:** 10.1039/d0ra02402c

**Published:** 2020-05-13

**Authors:** D. Triyono, S. N. Fitria, U. Hanifah

**Affiliations:** Department of Physics, Faculty of Mathematics and Natural Sciences (FMIPA), Universitas Indonesia Depok 16424 Indonesia djoko.triyono@ui.ac.id

## Abstract

Bulk-phase polycrystalline La_1−*x*_Bi_*x*_FeO_3_ (*x* = 0.1, 0.2, 0.3, 0.4, and 0.5) ceramics were prepared by citric sol–gel and sintering methods. The structural, morphological, and electrical properties of the resulting sol–gel solutions were investigated using various techniques. In an X-ray diffraction analysis, all samples crystallized in the orthorhombic structure with the *Pbnm* space group and showed an increase in lattice constant with increasing Bi content which was also confirmed by vibrational analysis. The sample surfaces and average grain sizes were examined by scanning electron microscopy. The grain distribution was non-uniform and the grain size increased with the increasing Bi content. The complex electrical conductivities and dielectric analyses of these materials were investigated as functions of frequency by impedance spectroscopy at various temperatures (75–200 °C). The frequency-dependent dielectric constant at each temperature increased with increasing Bi content. A Jonscher's power law analysis revealed that the AC and DC conductivities arose by completely different mechanisms. The temperature dependence and dielectric relaxation of the DC conductivity satisfied the Arrhenius law and decreased with increasing Bi content. The activation energy ranged from 0.20 to 0.45 eV and was similar in the conduction and relaxation mechanisms, indicating that both transport mechanisms were based on hopping phenomena. We believe that lowering the activation energy will help with the optimization of constituents as promising candidates in novel materials for future electrocatalysts.

## Abstract 2

Bulk-phase polycrystalline La_1−*x*_Bi_*x*_FeO_3_ (*x* = 0.1, 0.2, 0.3, 0.4, and 0.5) ceramics were prepared by citric sol–gel and sintering methods.

## Graphical abstract

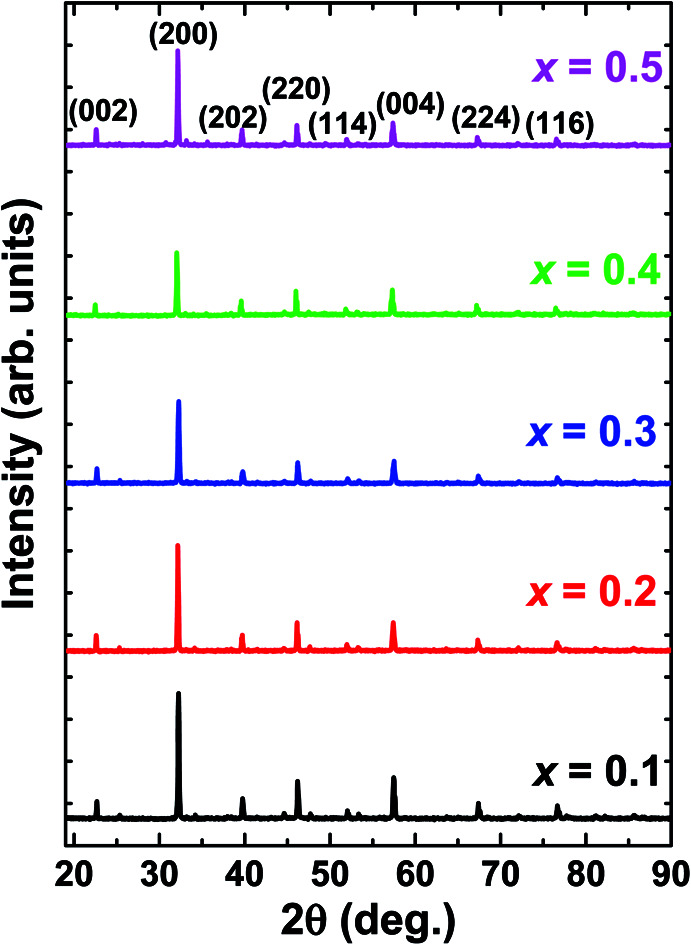

## 1. Introduction

ABO_3_ perovskites are well known as promising photocatalysts and for their excellence in electric conductive properties.^1,2^ LaFeO_3_ is a perovskite compound used as an ionic electronic conductor in photocatalysis.^3^ A few papers were published about LaFeO_3_ as having catalytic activity, because of its high stability, non-toxicity, and small band gap energy.^4^ However, the catalytic activity of pure LaFeO_3_ remains low because the oxygen evolution kinetics on the surface are sluggish and the charge transport is poor.^5^ Modifying LaFeO_3_ with monovalent and/or divalent ions at the La-sites and with transition metal ions at the Fe-sites can increase the oxygen ionic conductivity and affect the magnetic properties and electrical conductivity of the material, respectively.^3,6^ The modification can also enhance the electrical parameters of LaFeO_3_, change its lattice structure, and improve its crystal stability.^6,7^ Earlier research has focused on modifying the La site.^7–9^ Sr substitution at the La sites of LaFeO_3_ (La_1−*x*_Sr_*x*_FeO_3_) enhances the electrical conductivity and induces a transition of the crystal structure from orthorhombic to rhombohedral at *x* ≥ 0.4.^7^ LaFeO_3_ with Pb substituted La sites (La_1−*x*_Pb_*x*_FeO_3_, *x* ≥ 0.1) typically behaves as a material with a colossal dielectric constant (≥10^4^), in which the relaxation and conduction mechanisms are mediated by hopping polarons.^8^ Na substitution on La-site of LaFeO_3_ (La_1−*x*_Na_*x*_FeO_3_) enhances the dielectric constant to up to 10^5^ at 100 Hz. The magnetization of La_1−*x*_Na_*x*_FeO_3_ reached 2.11 emu per g at 10 kOe at room temperature.^9^

Some studies have been done for Bi substituted in La site of LaFeO_3_.^10–19^ Bi-doped was reported causing noticeable improvement on the conductivity and electrochemical performance.^10,11^ In this, Bi-doped improved the electrochemical performance and reduced the interfacial polarization resistance from 1 to 0.1 Ω cm^2^ at 700 °C.^10^ Meng, *et al.*^11^ reported that Bi ion plays more importance role on electrochemical properties than microstructure and also can improves conductive properties in LaFeO_3_-based. Li *et al.*^12^ displayed that La_1−*x*_Bi_*x*_FeO_3_ powders (0 ≤ *x* ≤ 0.2) showed significantly effort to enhance the activity and effective approaching to optimize the crystal structure, enhance the surface oxygen vacancy, change the valence states of ions of the perovskite electrocatalysts, especially in La_0.85_Bi_0.15_FeO_3_ (the lowest ORR inset potential, the largest ORR kinetic current density, the lowest Tafel slope, and the optimal electron-transfer number). Ahmed *et al.*^13^ also reported that Bi substitution on La_1−*x*_Bi_*x*_FeO_3_ (0 ≤ *x* ≤ 0.2) caused the increasing of magnetization with Bi content and showed weak ferromagnetism influenced by both particle size and morphology at room temperature. Rangi *et al.*^14^ reported that Bi_1−*x*_La_*x*_FeO_3_ with *x* = 0.5 showed the best value of magnetization due to mixed structural phase. Rusakov *et al.*^15^ reported that replace La^3+^ with Bi^3+^ showing better structural stability than modification with another rare-earth element. Chen *et al.*^16^ studied that the thermal stability of BiFeO_3_ was greatly enhanced by substitution with LaFeO_3_. Khalek *et al.*^17^ investigated the structural change from orthorhombic (*Pbnm*) to rhombohedral (*R*3*c*) phases for Bi_1−*x*_La_*x*_FeO_3_ (*x* = 0.5 and 0.75), which is related to anomalous phenomenon dielectric constant at room temperature in the microwave region exhibited. Some theoretical studies about structural changes of Bi_1−*x*_La_*x*_FeO_3_ with *x* = 0 to 1 have been investigated. By using first principle calculation, Kaczkowski^18^ reported the structural transition from rhombohedral to orthorhombic structure of Bi_1−*x*_La_*x*_FeO_3_ was reached at *x* = 0.32. Another previous study by Arnold^19^ showed that lanthanum substitution lead the change to orthorhombic symmetry is coupled with a dramatic decrease in the lattice parameter, *c*. Previous report by Mao *et al.*^20^ displayed that single phase Eu and Sr- and Co-doped BiFeO_3_ is effective to enhance the multiferroics properties and minimize the leakage current for practical applications.

However, to our knowledge, La substitution with such tetravalent cations has not been widely explored. Therefore, a meticulous study on the factors influencing the structural changes and electrical mechanism of Bi-substitution on LaFeO_3_ is an interesting proposition. In this work, Bi ions were inserted into the La sites of LaFeO_3_ by a sol–gel method. The structural, morphological, and electrical characteristics of the La_1−*x*_Bi_*x*_FeO_3_ ceramics were investigated. The crystal structure and morphological characteristics were studied by X-ray diffraction (XRD) and scanning electron microscopy (SEM). The frequency and temperature dependences on the dielectric and electrical mechanisms in the La_1−*x*_Bi_*x*_FeO_3_ system were then studied by alternating current (ac) impedance analysis. The results are discussed below.

## 2. Experimental details

La_1−*x*_Bi_*x*_FeO_3_ nanoparticles were prepared by citric sol–gel route, as reported by Fitria, *et al.*^21^ The raw materials were dissolved in citric acid monohydrate at 120 °C for 8 h using a stirring magnetic mixer. The solution was dried and calcined to obtain the nano-crystalline powder form. In preparation for the impedance spectroscopy (IS) measurements, the powdered sample was ground and pressed into pellet/bulk and then sintered to form the bulk-phase ceramics. Finally, the ceramics were annealed at 900 °C for 6 h to enhance the crystallization and homogeneity of their microstructure.

The lattice structure of the samples was determined by XRD (PANalytical X'pert Pro) with Cu-Kα radiation. The data were collected in the range of 20° to 2*θ*–90°, with a scanning step of 0.04°. The morphology of the prepared samples was analysed by SEM (QUANTA 650). The surface of all samples was gold-coated prior to surface observation. IS measurements were carried out in the temperature range of 75–200 °C while sweeping the frequency from 100 Hz to 1 MHz. The IS instrument was an RLC meter (FLUKE-PM 6303), and the circuit configuration was a single parallel resistance–capacitance configuration. The vibrational properties of the ceramics were measured using Raman scattering spectroscopy (THERMO SCIENTIFIC: DXR2 Raman Microscope) with a laser excitation wavelength of 532 nm.

## 3. Results and discussion

### 3.1 XRD analysis

**Fig. 1:**
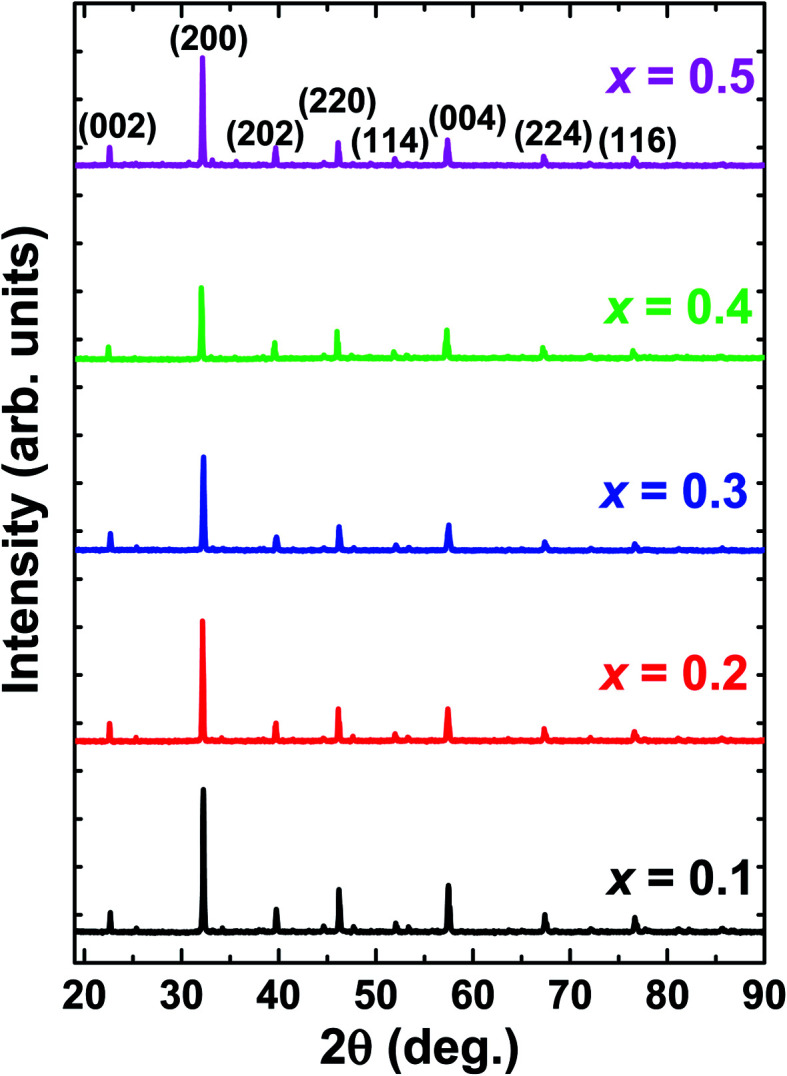
XRD patterns of La_1−*x*_Bi_*x*_FeO_3_ ceramics (*x* = 0.1, 0.2, 0.3, 0.4, and 0.5).

Fig. 1 depicts the X-ray diffraction (XRD) patterns of La_1−*x*_Bi_*x*_FeO_3_ ceramics under study. All samples have been identified with well-defined diffraction peaks of (002), (200), (202), (202), (114), (004), (224), and (116). There is no evidence of any secondary phase/impurity could be detected up to the error detection limit of XRD. Based on previously reported,^12–19^ pure LaFeO_3_ and BiFeO_3_ parent compound has the orthorhombic and rhombohedral crystal structure, respectively. However, our samples show no additional impurity peaks indicating that Bi^3+^ ions successfully occupied LaFeO_3_ perovskite lattice.

**Fig. 2:**
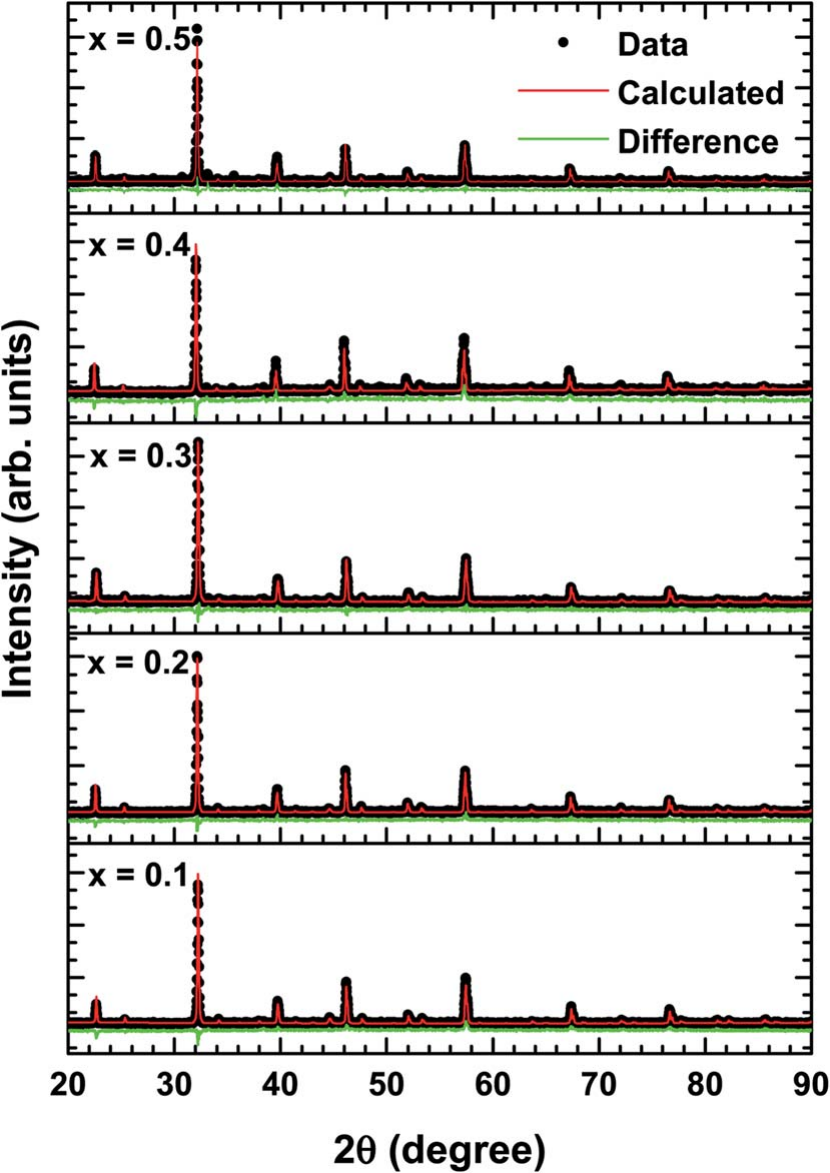
Rietveld refinement results of XRD patterns of La_1−*x*_Bi_*x*_FeO_3_ ceramics (*x* = 0.1, 0.2, 0.3, 0.4, and 0.5).

The structural changes in term of lattice parameters, bond angle and bond length was obtained from refinement results. We refined the structural parameters of the samples using Fullprof 2k and VESTA software. Fig. 2 shows the Le-bail fitting as Rietveld refinement results of XRD pattern from the prepared La_1−*x*_Bi_*x*_FeO_3_ samples. We determine black dot as the experimental data, red lines as fitting results, and the green line as the difference between the experimental data and calculation. The difference pattern exhibited a good agreement with fairly accepted value *χ*^2^ as written in Table 2. The XRD patterns of all samples studied are in good agreement with LaFeO_3_-parent compound in the standard from JCPDS 96-1526451 data base. All samples exhibited the same crystalline perovskite structure, namely, the orthorhombic lattice belonging to the *Pbnm* space group, without any secondary phase. There are no additional peaks corresponding to the absence of secondary phase.

The average crystallite size calculated using Debye–Scherrer formula:^13^
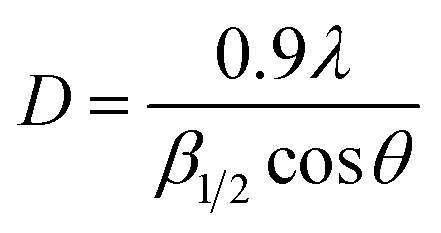
where *λ* is the wavelength of Cu-Kα (1.5406 Å), *β* is full width at half maximum (FWHM) intensity in degree, and *θ* is Bragg angle. The average crystallite size increase with increasing Bi-content.

The final crystallographic parameters are summarised in Table 1. As the Bi content increased, the lattice parameters changed slightly but with a noticeable overall effect on the cell volume. The different ionic radii of the different cations probably contracted the lattice by distorting the FeO_6_ octahedra.^6^ However, the changes in the present case were minor because the ionic radii of La^3+^ (112 pm) and Bi^3+^ (117 pm) are similar, so the Bi-substitution at La sites only slightly increased the lattice parameters. This inference can be confirmed by observing the tolerance factor (an indicator of structural distortion) and the geometrical parameters.

**Table 1:** Crystallographic parameters of the La_1−*x*_Bi_*x*_FeO_3_ ceramics (*x* = 0.1, 0.2, 0.3, 0.4, and 0.5), obtained by the FullProf 2k programme

	Parameters				
	*a* (Å)	*b* (Å)	*c* (Å)	Volume (Å^3^)	Crystallite size (nm)
*x* = 0.1	5.5499(8)	5.5654(2)	7.8536(8)	242.58(4)	256.49(3)
*x* = 0.2	5.5525(4)	5.5654(3)	7.8548(0)	242.73(1)	259.02(3)
*x* = 0.3	5.5516(4)	5.5709(8)	7.8587(0)	243.05(4)	273.18(8)
*x* = 0.4	5.5536(8)	5.5703(2)	7.8605(4)	243.17(2)	396.47(1)
*x* = 0.5	5.5636(3)	5.5814(9)	7.8844(9)	244.84(0)	525.61(2)

The tolerance factor *t* is calculated as follows:^19^
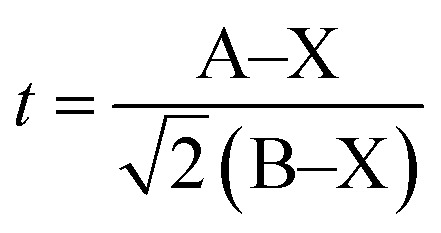
where A–X is the bond length of A-site cation and the X-site anion (in this case La/Bi–O), and B–X is the bonding distance of B-site cation and the X-site anion (in this case Fe–O). The bond lengths, other geometrical parameters, and the calculated tolerance factor are tabulated in Table 2. The obtained tolerance factors indicate that the structural distortion increased with increasing Bi content. The tolerance factor was an increasing function of the ionic radius of the A-site cation, as confirmed by the decreasing FeO_6_ tilting angle (see Table 2), indicating a reduced driving force of the octahedral tilting.

**Table 2:** Geometrical parameters characterizing the crystal structure of La_1−*x*_Bi_*x*_FeO_3_ ceramics (*x* = 0.1, 0.2, 0.3, 0.4, and 0.5)

Parameters	*x* = 0.1	*x* = 0.2	*x* = 0.3	*x* = 0.4	*x* = 0.5
**Atomic position**					
*La/Bi*					
*x*	0.9930	0.9930	0.9930	0.98996	0.99338
*y*	0.0297	0.0297	0.0297	0.02491	0.02301
*z*	0.25	0.25	0.25	0.25	0.25

**Fe**					
*x*	0	0	0	0	0
*y*	0.5	0.5	0.5	0.5	0.5
*z*	0	0	0	0	0

**O1**					
*x*	0.719	0.719	0.719	0.719	0.719
*y*	0.302	0.302	0.302	0.302	0.302
*z*	0.029	0.029	0.029	0.029	0.029

**O2**					
*x*	0.08	0.08	0.08	0.08	0.08
*y*	0.485	0.485	0.485	0.485	0.485
*z*	0.25	0.25	0.25	0.25	0.25

**Wickoff position**					
La/Bi	4c	4c	4c	4c	4c
Fe	4b	4b	4b	4b	4b
O1	4c	4c	4c	4c	4c
O2	8d	8d	8d	8d	8d

**Bond angle (°)**					
Fe–O2–Fe	154.07(6)	154.08(3)	154.09(1)	154.08(8)	154.12(0)
Fe–O1–Fe	157.07(0)	157.07(0)	157.07(0)	157.06(1)	157.06(1)

**Bond length (Å)**					
La/Bi–O1 (m)	2.4500(7)	2.4505(0)	2.4516(7)	2.4302(9)	2.4401(0)
La/Bi–O1 (l)	2.6929(3)	2.6934(5)	2.6945(6)	2.7124(3)	2.7145(2)
La/Bi–O2 (s)	2.3828(6)	2.3839(5)	2.3835(9)	2.3986(3)	2.4078(1)
La/Bi–O	2.5086(2)	2.5093(0)	2.5099(4)	2.5137(8)	2.5208(1)
Fe–O1 (l)	2.0866(6)	2.0869(8)	2.0882(3)	2.0883(4)	2.0924(0)
Fe–O1 (s)	1.9231(1)	1.9237(0)	1.9241(4)	1.9245(3)	1.9281(5)
Fe–O2 (m)	2.0147(3)	2.0150(4)	2.0159(8)	2.0165(0)	2.0224(8)
Fe–O	2.0081(6)	2.0085(7)	2.0094(5)	2.0097(9)	2.0143(4)
Tolerance factor	0.8833	0.8834	0.8837	0.8844	0.8849

***R*-factors**					
*R*_p_	5.60	5.89	6.56	8.36	6.06
*R*_wp_	7.50	7.59	8.52	12.5	8.17
*R*_e_	6.10	6.57	7.40	7.36	6.76
*χ* ^2^	1.51	1.33	1.33	2.89	1.46
Average tilt angle 〈*φ*〉 (°)	14.915(1)	14.915(2)	14.912(4)	14.912(4)	14.906(1)

### 3.2 Vibrational analysis

**Fig. 3:**
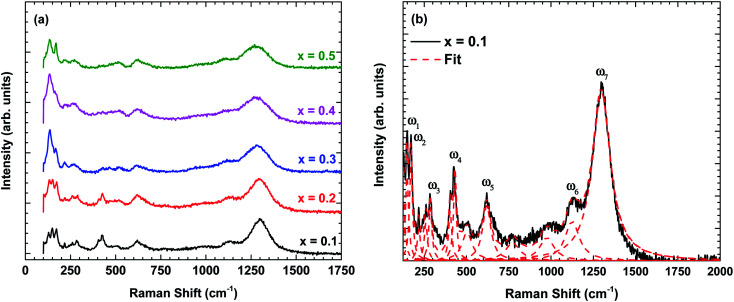
(a) Raman scattering spectra of La_1−*x*_Bi_x_FeO_3_ ceramics (*x* = 0.1, 0.2, 0.3, 0.4, and 0.5) at room temperature and (b) the fitting results displayed for *x* = 0.1 using standard Lorentzian profile.

Fig. 3 shows the Raman scattering spectra of La_1−*x*_Bi_*x*_FeO_3_ ceramics (*x* = 0.1, 0.2, 0.3, 0.4, and 0.5). The Raman phonon modes were fitted with a standard Lorentzian profile and the background was fitted with the standard Gaussian profile.^23^ Some specific Raman phonon modes of each sample from fitting results are tabulated in Table 3. The Raman-active phonons of these samples are in good agreement with previous researches.^24,25^

**Table 3:** Frequency and symmetry assignments of some specific Raman-active phonon modes observed La_1−*x*_Bi_*x*_FeO_3_ ceramics (*x* = 0.1, 0.2, 0.3, 0.4, and 0.5) at room temperature. All values are measured in cm^−1^

	*x* = 0.1	*x* = 0.2	*x* = 0.3	*x* = 0.4	*x* = 0.5	Symmetry
*ω*_1_	148.4	147.5	145.2	144.7	143.8	A_g_
*ω*_2_	171.9	171.2	169.0	170.1	169.2	A_g_
*ω*_3_	286.1	286.4	283.6	283.0	262.1	A_g_
*ω*_4_	431.3	427.7	428.7	426.7	428.6	A_g_
*ω*_5_	619.6	619.3	619.0	619.2	618.8	B_1g_
*ω*_6_	1125	1119	1115	1103	1103	Second-order
*ω*_7_	1297	1297	1283	1277	1277	Second-order

Following previous reports,^24,25^ the Raman phonon modes below 200 cm^−1^ are related to La/Bi-vibration with A_g_ symmetry. The Raman phonon modes between 250–350 cm^−1^ are associated with the tilting of octahedra corresponding to A_g_ symmetry. Finally, the Raman phonon modes between 400–500 cm^−1^ are assigned to the Jahn–Teller distortion with A_g_ symmetry, whereas the modes between 500–750 cm^−1^ corresponds to the symmetric stretching of FeO_6_ octahedra with B_1g_ symmetry. Additional phonon modes between 700–1000 cm^−1^ are possibly related to Franck–Condon phonon modes.^26^ Another Raman phonon mode observed above 1000 cm^−1^ is related to second-order scattering.^27^

Now, we turn to explain the effect of Bi content on the phonon characteristics, *i.e.*, Raman modes intensity, linewidth, and wavenumber. As shown in Fig. 3(a), the Raman modes intensity is reduced and linewidth becomes more broadens with increasing of Bi content indicating the increase in lattice disorder. In other words, Bi substitution changes the lattice constant, causing the change in Jahn–Teller distortion.^28^ This is consistent with the observation of an increase in the calculation tolerance factor by X-ray diffraction analysis. Besides, some specific Raman phonon modes are denoted in Fig. 3(b) and summarized in Table 3. The Raman phonon modes tend to shift to the lower wavenumber confirming the lattice constant and bond length increased with increasing Bi content which is consistent with X-ray diffraction analysis (Tables 1 and 2).

### 3.3 Morphological characterisation

**Fig. 4:**
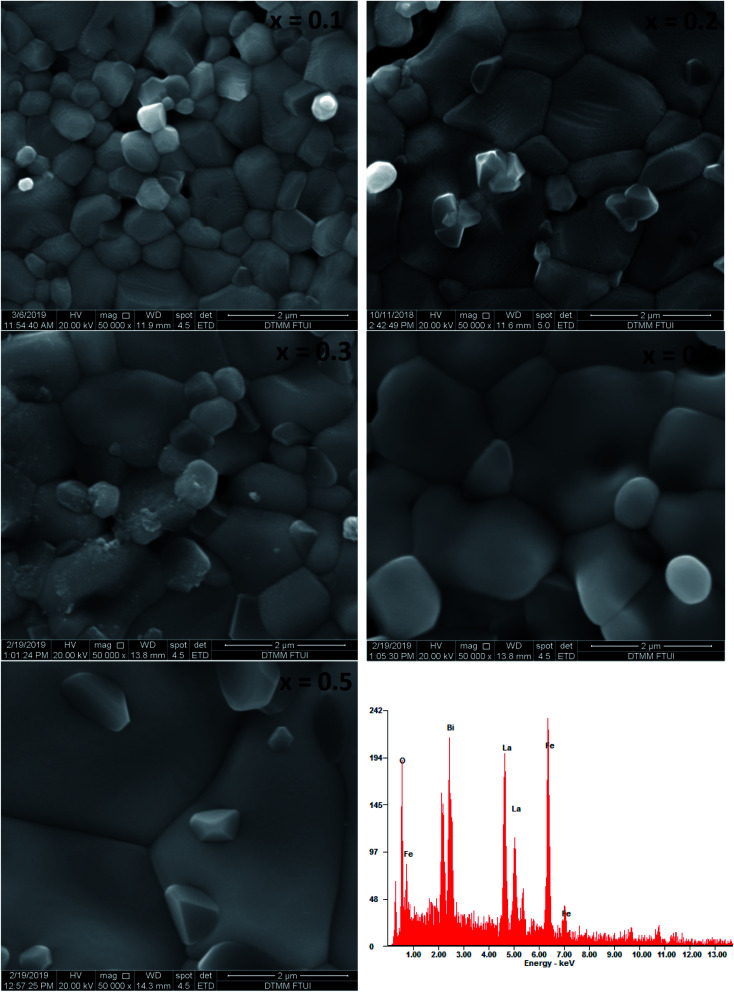
SEM images of annealed La_1−*x*_Bi_*x*_FeO_3_ ceramics (*x* = 0.1, 0.2, 0.3, 0.4, and 0.5) and the energy dispersive X-ray spectrum of the La_0.5_Bi_0.5_FeO_3_ ceramic.

Fig. 4 shows the SEM micrographs of the prepared samples. The samples show a typical polycrystalline microstructure with a larger non-uniform grain distribution separated by grain boundaries throughout the samples. The average grain size was estimated as 0.7–2.2 μm and increased with increasing Bi content which is in the similar trend with crystallite size calculated. This phenomenon might be due to the difference of optimum heating temperature of LaFeO_3_ and BiFeO_3_ where Bi has lower the optimum sintering temperature in La_1−*x*_Bi_*x*_FeO_3_, which lead the cells agglomerate and generate the larger grain size.^12^ In the typical energy dispersive X-ray spectroscopy pattern of La_0.5_Bi_0.5_FeO_3_ ceramic, all elemental compositions (La, Bi, Fe, and O) presented in their respective molar concentrations during the annealing process.

### 3.4 Electrical impedance analysis

**Fig. 5:**
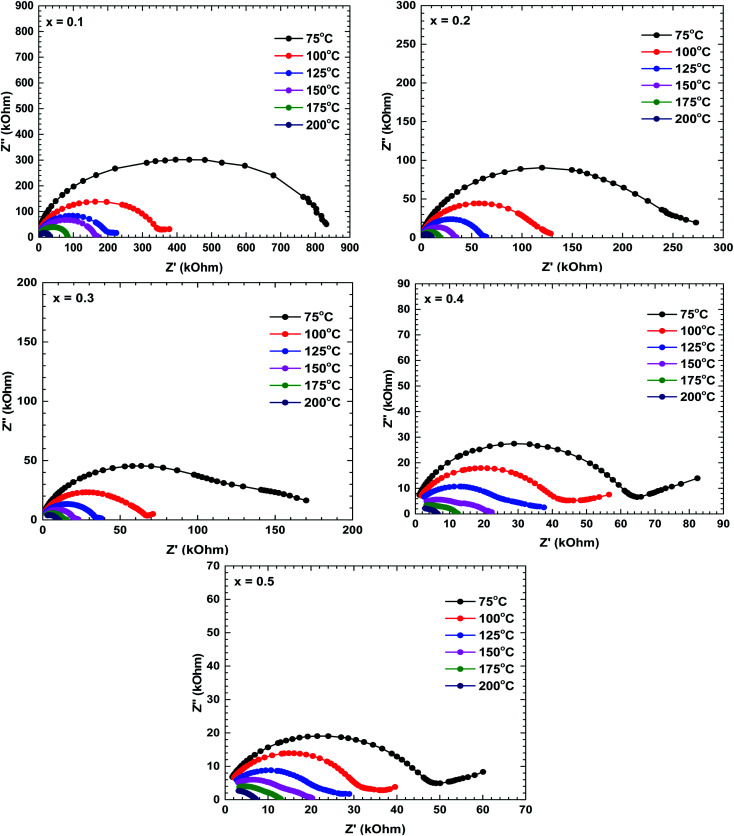
Nyquist plots of the La_1−*x*_Bi_*x*_FeO_3_ ceramics (*x* = 0.1, 0.2, 0.3, 0.4, and 0.5).

Fig. 5 shows the Nyquist plots (*Z*′′ *vs. Z*′) of all prepared samples. The electrical analysis for un-substituted sample has been reported in our previous work.^28^ These plots are characterised by double semicircular arcs off-centred from the real axis. The high- and low-frequency semicircular arcs represent the grain and grain boundary contributions, respectively. The asymmetry and depressed arc indicates a non-Debye type relaxation.^29,30^ The diameter of the semicircular arcs, which is equivalent to the impedance value of the constituent materials, decreased with increasing temperature, indicating semiconducting behaviour of these materials.^30^

The impedance decreased with increasing temperature, suggesting increased movement of the charge carriers involved in the conduction mechanism.^29^ The increase or decrease of semicircular arcs can anticipate the dominant roles of different conduction mechanisms. At room temperature, the low-frequency semicircular arcs of the La_1−*x*_Bi_*x*_FeO_3_ ceramics were larger than the high-frequency arcs, implying that the grain boundary contribution dominated over the grain contribution. As the temperature increased, the emergence of semicircles in the high-frequency region implied an increasing grain effect; eventually, the grain contribution dominated over the grain boundary contribution.^22^ Additionally, the impedance decreased with increasing Bi content, indicating that the Bi substituents at La sites played an important role in the electrical conductivity of this La_1−*x*_Bi_*x*_FeO_3_ ceramics system.

**Fig. 6:**
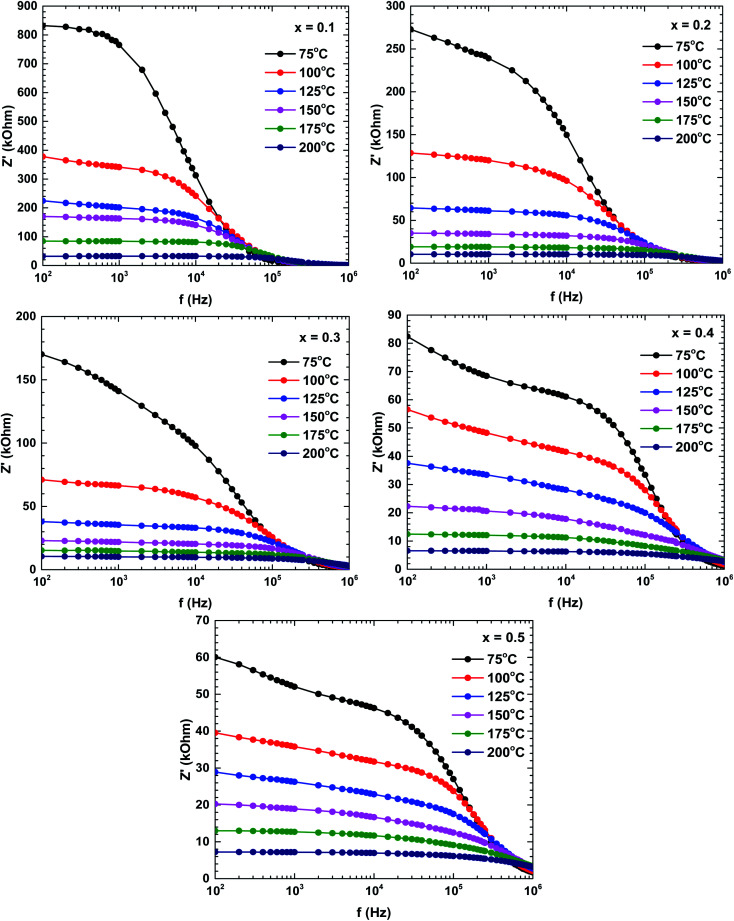
Bode plots (real impedance) of the La_1−*x*_Bi_*x*_FeO_3_ ceramics (*x* = 0.1, 0.2, 0.3, 0.4, and 0.5).

Fig. 6 shows the real impedance *versus* frequency relationships (Bode plots) of the samples prepared at different temperatures. In the low-frequency region, the real impedance was higher at lower temperatures than at higher temperatures, suggesting an increase in the electrical conductivity with increasing temperature. The real impedance also decreased with increasing frequency. The real impedances merged in the high-frequency region, possibly because the barrier reduction at high temperature was compensated by the release of space charges.^29,30^

**Fig. 7:**
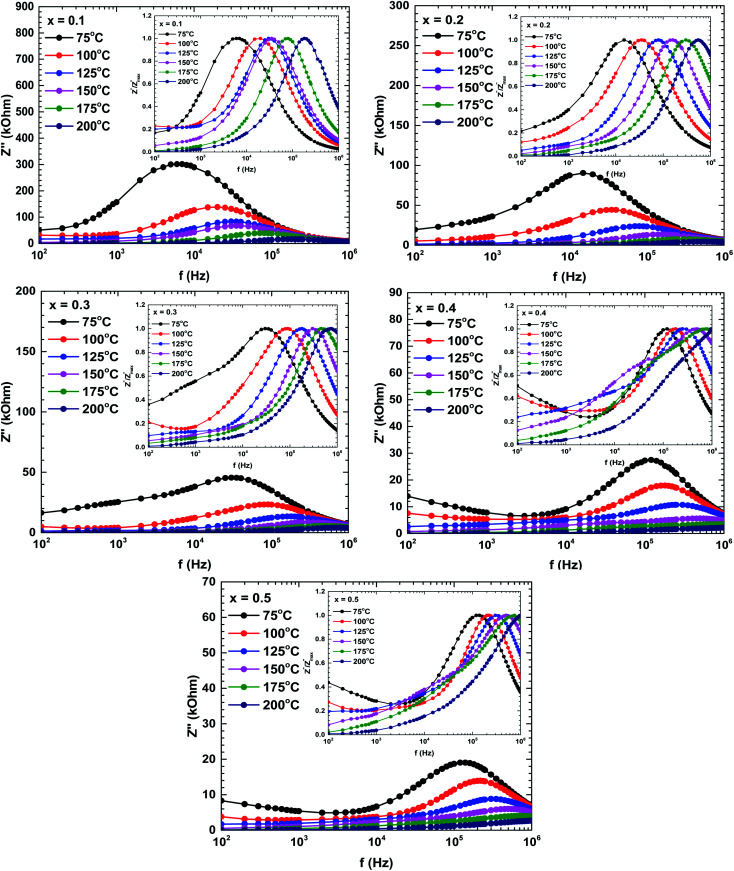
Bode plots (imaginary impedances) of the La_1−*x*_Bi_*x*_FeO_3_ ceramics (*x* = 0.1, 0.2, 0.3, 0.4, and 0.5). The inset in each figure describes the normalized spectra of the imaginary impedance 
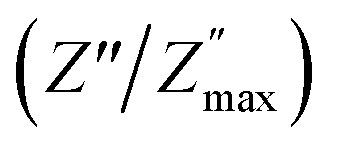
 at different temperatures.

Fig. 7 plots the imaginary impedance *versus* frequency (Bode plots) of the samples prepared at different temperatures. Regardless of temperature, the imaginary impedance increased with frequency up to some maximum and then decreased at higher frequencies. However, the peak frequency increased with temperature and also broadened, indicating a thermal relaxation mechanism.^29^ This mechanism is plausibly caused by the immobile species at lower temperatures and by defects at higher temperatures.^29,31^

The shifting peaks toward the high-frequency region indicate a decrease in relaxation time as the temperature increased. The relaxation time *τ* satisfied the following Arrhenius law:^29–31^
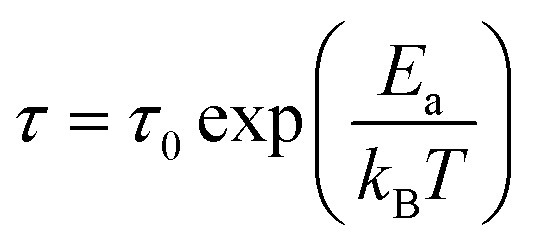
where *τ*_0_ is the characteristic relaxation time, *E*_a_ is the activation energy of the relaxation process, *k*_B_ is the Boltzmann constant, and *T* is the temperature.

The activation energy of the relaxation mechanism in each sample is tabulated in Table 4. The activation energy decreased with increasing Bi content, indicating increased hopping of the charge carrier concentration between the neighbouring lattice sites.^32^

**Table 4:** Activation energy of the La_1−*x*_Bi_*x*_FeO_3_ ceramics (*x* = 0.1, 0.2, 0.3, 0.4, and 0.5)

	*x* = 0.1	*x* = 0.2	*x* = 0.3	*x* = 0.4	*x* = 0.5
*E*_a_ (eV)	0.43	0.41	0.35	0.25	0.23
*τ*_0_ (×10^−10^ s)	2.36	0.13	0.36	3.40	6.22

To interpret the dielectric relaxation, the normalized spectra of the imaginary impedance in each sample are presented in the insets of Fig. 7. All normalized spectra were non-overlapping and scaled to the multiple master curve, indicating that the relaxation dynamics were temperature-dependent and localized.^33^ It was also noticed that all spectra shifted to higher frequencies with increasing temperature.

**Fig. 8:**
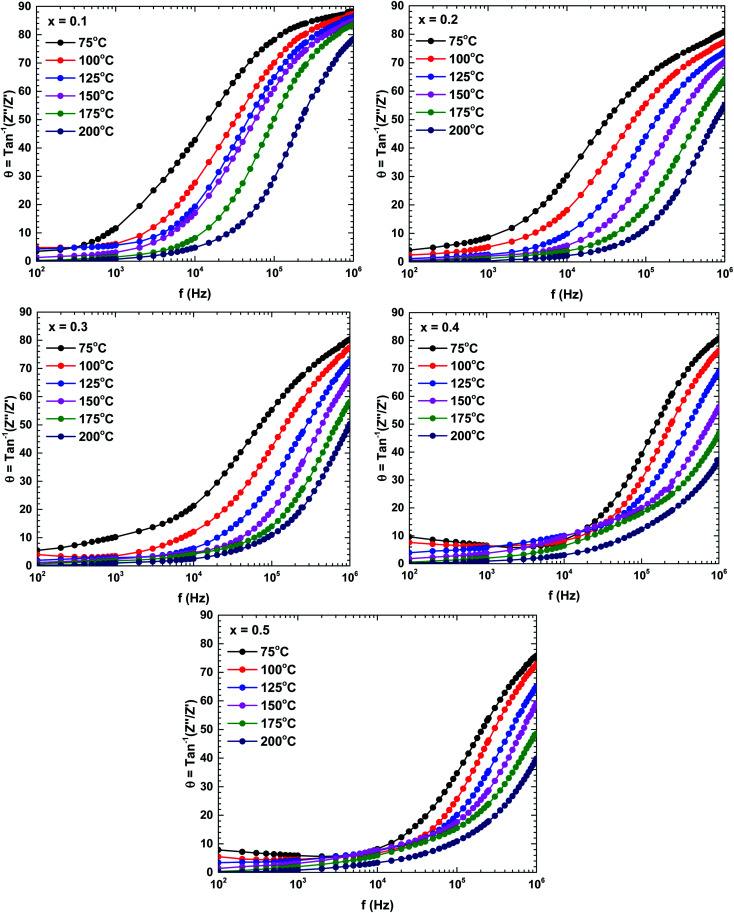
Bode plots (phases) of the La_1−*x*_Bi_*x*_FeO_3_ ceramics (*x* = 0.1, 0.2, 0.3, 0.4, and 0.5).

To determine the contributions of the grains and grain boundaries in the conduction process, Fig. 8 presents Bode plots of the phase angles of the samples prepared at different temperatures. Two peaks, one each in the high-and low-frequency regions, indicate a mixing of the grain and grain boundary contributions in the electrical transport mechanism.^34^

**Fig. 9:**
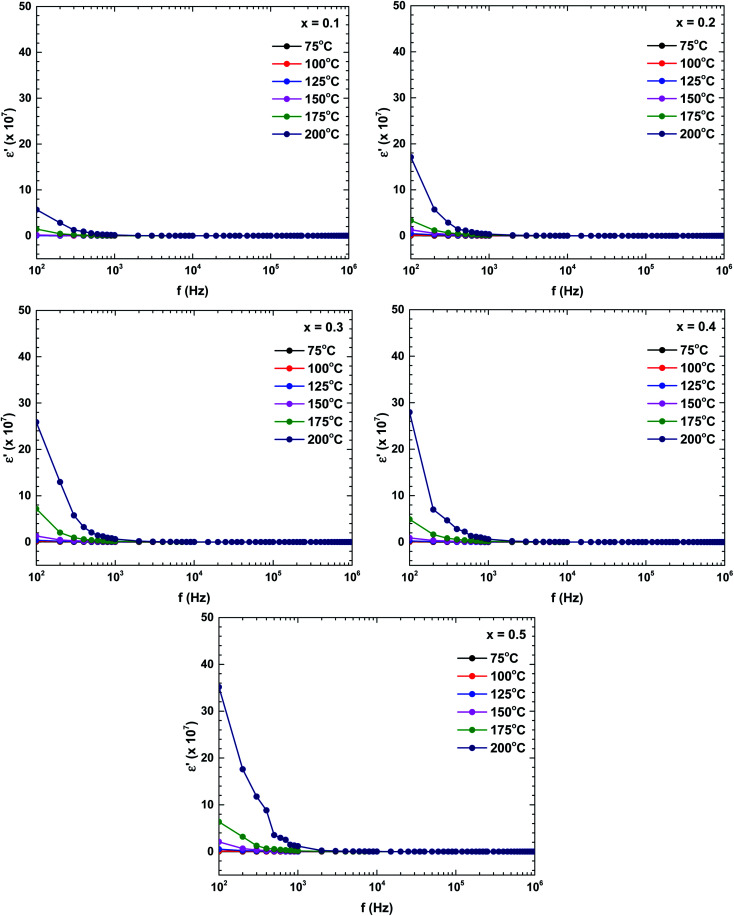
Frequency and temperature dependencies of the dielectric constant of the La_1−*x*_Bi_*x*_FeO_3_ ceramics (*x* = 0.1, 0.2, 0.3, 0.4, and 0.5).

Fig. 9 plots the dielectric constants of the samples as functions of frequency at various temperatures. The dielectric constant changed with both frequency and temperature. The dielectric constant was relatively high (indicating dispersion) at a lower frequency, and decreased with increasing frequency. The dielectric constant at low frequency also strongly increased with increasing temperature. The dielectric behaviour was dominated by a polarisation process originating from the grain boundary contribution. In the low-frequency region, the charge carriers accumulated in the grain boundary and the hopping process required more energy than at higher frequencies, boosting the dielectric constant.^35^ Moreover, the dielectric constant was an increasing function of Bi content. Those dielectric constant value significantly increased compared with the pure-LaFeO_3_ compound.^28,36^

**Fig. 10:**
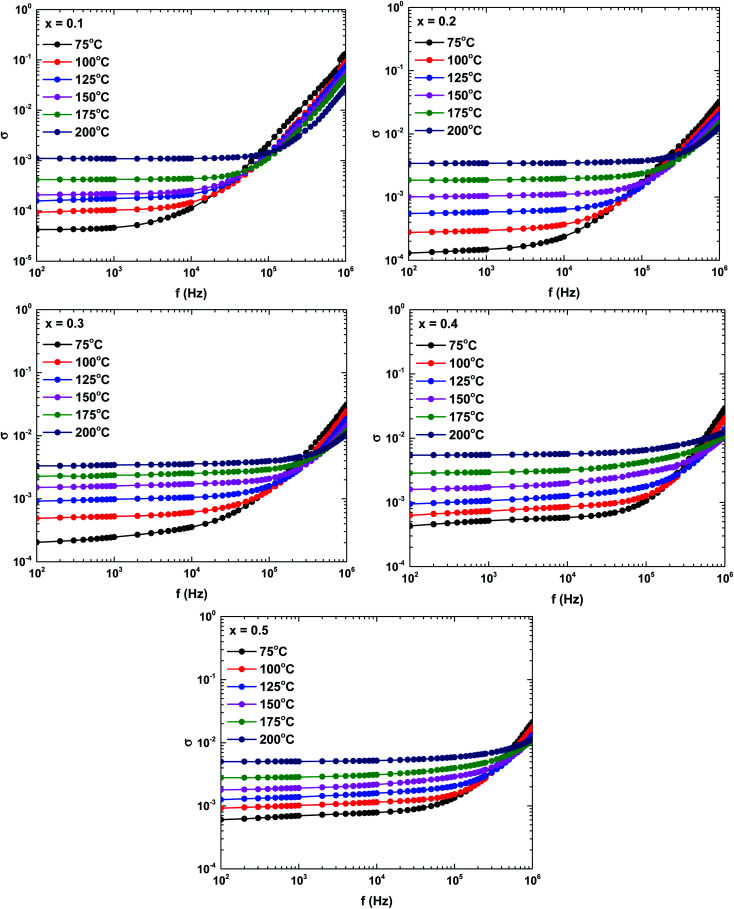
Frequency and temperature dependencies of the complex conductivities of the La_1−*x*_Bi_*x*_FeO_3_ ceramics (*x* = 0.1, 0.2, 0.3, 0.4, and 0.5).

Fig. 10 shows the frequency dependence of the complex conductivity in the samples prepared at different temperatures. The complex conductivity spectra were divisible into two parts. The first part reflected the frequency-independent behaviour of the conductivity in the low-frequency region, the so-called DC conductivity. The second part was attributed to the AC conductivity, which is an increasing function of frequency. According to Koop's theory, the AC conductivity represents the dispersion in the high-frequency region, which is attributable to the highly conductive grains and the highly resistive grain boundaries.^35^ The increased grain conductivity at high frequencies is possibly caused by intensified hopping of the charge carrier mechanism. Meanwhile, the increased AC and DC conductivities at higher temperatures might be attributable to the increased tunnelling probability of charge carriers.^35,37^ Overall, the frequency dependence of the complex conductivity satisfied Jonscher's power law:^37^*σ*(*ω*) = *σ*_DC_ + *Aω*^*s*^where *σ*_DC_ is the DC conductivity, *ω* is the (measured) angular frequency, the exponent *s* is a temperature-dependent constant, and *A* is a constant that determines the strength of the polarization, and is strongly dependent on the temperature and composition of the samples. Overall, the second term on the right-hand-side of Jonscher's power–law equation represents the frequency-dependent (AC) conductivity region.^37^ The AC and DC conductivities are considered to arise from completely different mechanisms, as explained below.

**Fig. 11:**
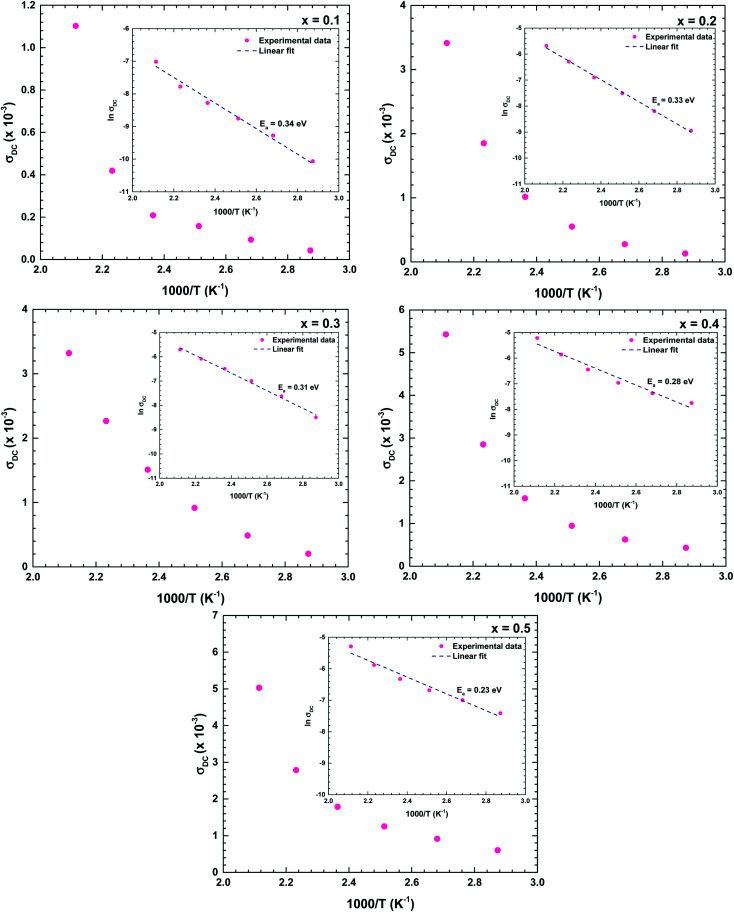
Temperature dependence of the DC conductivity of the La_1−*x*_Bi_*x*_FeO_3_ ceramics (*x* = 0.1, 0.2, 0.3, 0.4, and 0.5). Insets describe the linear ln(*σ*_DC_) *vs.* 1/*T* plots, which satisfy the Arrhenius law.

Fig. 11 shows the temperature dependences of the DC conductivities of the prepared samples. The DC conductivity increased with increasing temperature, confirming a thermally activated transport process in the conduction mechanism. As the conduction mechanism satisfies the Arrhenius law, the activation energy can be obtained from the Arrhenius equation as follows:^34,37,38^
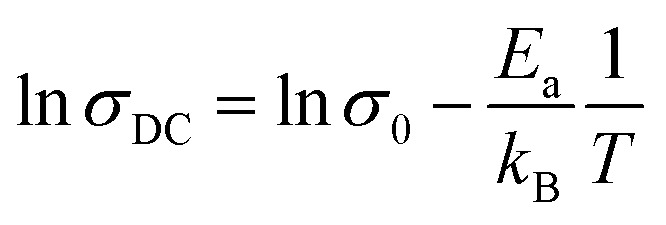
where the symbols have their previously defined meanings. The insets of Fig. 11 are the Arrhenius plots of DC conductivity *versus* temperature.

The obtained activation energies ranged from 0.20 to 0.35 eV and decreased with increasing Bi content of the ceramic. The Bi substituents increased the lattice volume, so the specimen responsible for conduction were more easily released; that is, required lower energy for mobility. The DC conductivity also increased with increasing Bi content, confirming that the Bi substituents at the La sites strongly affected the conductivity of the La_1−*x*_Bi_*x*_FeO_3_ ceramic oxides. The obtained activation energies were similar to those of a dielectric relaxation analysis, indicating that the electrical transport arose from the hopping mechanism.^39^ The activation energies of all samples satisfied 0.20 < *E*_a_ < 1.0 eV, indicating a conduction mechanism dominated by p-type polaron hopping.^6,8^ Lowering the activation energy following by increasing in high conductivity makes our as-synthesize La_1−*x*_Bi_*x*_FeO_3_ a promising candidate for catalyst material applying in electrode surface for electrochemical reactions.^20^ With required lows activation energy as inhibitor, the kinetics energy could be increased, so that improve the large amount of electrochemical reaction occurring in the system. Consequently, this study could have the benefit of understanding and further development in electrochemical applications.

**Fig. 12:**
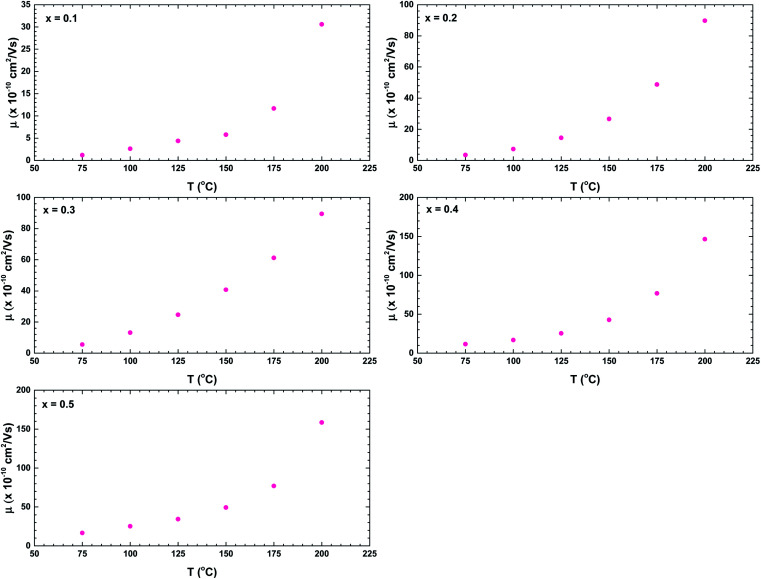
Temperature dependences of the charge carriers mobilities of the La_1−*x*_Bi_*x*_FeO_3_ ceramics (*x* = 0.1, 0.2, 0.3, 0.4, and 0.5).

The increased conductivities at higher frequencies are attributable to the higher mobility of charge carriers at higher temperatures than at lower temperatures.^35^ The carrier mobility *μ* is calculated as follows:^35,40^
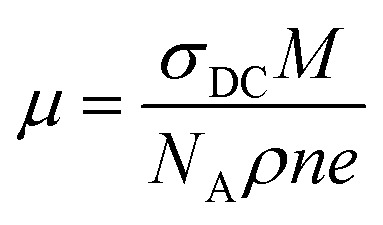
where *M* is the molecular weight of the sample, *ρ* is the calculated bulk density, *n* is the number of charge carriers involved, and *e* is the elementary charge. The obtained results are presented in Fig. 12. The mobility of the charge carriers increased with temperature and with Bi content (which creates the charge carrier mobility) in the system. The mobility was smaller than that of electrons and holes,^32^ excluding electron and/or hole mobility from the DC conduction mechanism. The mobility range suggested a role for small polarons in the conduction mechanism, which arises by electron-trapping in the lattice when adjacent atoms or ions are displaced.^32,33^

**Fig. 13:**
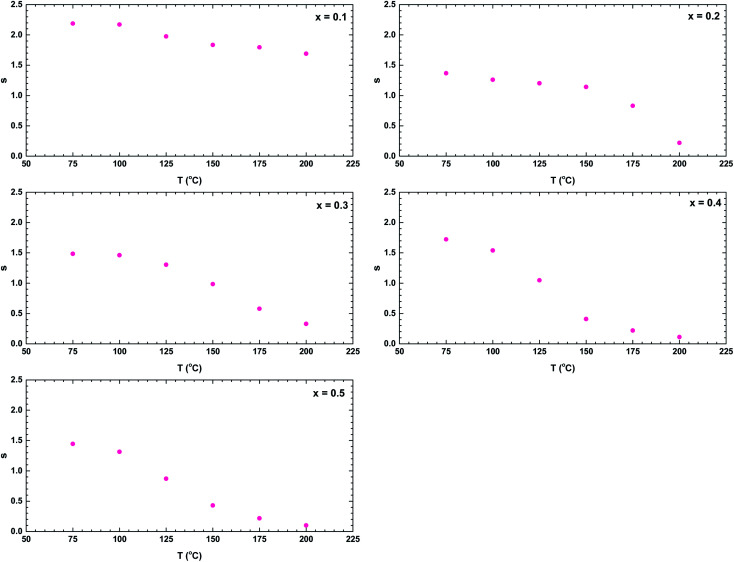
Temperature dependence of the frequency exponent *s* of the La_1−*x*_Bi_*x*_FeO_3_ ceramics (*x* = 0.1, 0.2, 0.3, 0.4, and 0.5).

Finally, we consider the AC conductivity region. The possible mechanism of the AC conductivity can be deduced from the temperature variation of the frequency exponent *s*.^40^Fig. 13 shows the temperature dependences of the parameter *s* for all samples. The value decreased with temperature, indicating a conduction mechanism dominated by correlated barrier-hopping.^40,41^ When *s* < 1, the hopping process jerks with translational motion, whereas *s* > 1 suggests a localised hopping that does not leave the neighbourhood.^38^ The ceramic with *x* = 0.1 exhibited a localised hopping process throughout the investigated temperature range. In the samples with higher Bi content, both hopping mechanisms were involved; accordingly, the Bi content played an important role in the hopping transformation in the La_1−*x*_Bi_*x*_FeO_3_ ceramics system. Over the investigated temperature range, the dominance of translational motion with a sudden hopping mechanism increased with increasing Bi content.

## 4. Conclusions

In this work, we successfully synthesised La_1−*x*_Bi_*x*_FeO_3_ ceramics by the sol–gel and annealing method. The lattice parameters and average grain size increased with increasing Bi content. The vibrational analysis confirmed the changes in Raman phonon characteristics with increasing Bi content confirming the increasing of lattice disorder which is in agreement with XRD analysis. Two semicircles in the impedance plots confirmed the contributions of both grains and grain boundaries in the electrical transport mechanism. The dielectric constant increased with increasing Bi content. The activation energies were similar in the electrical conduction and relaxation mechanisms, indicating a common electrical transport mechanism. Also, the activation energy ranged from 0.20 to 0.45 eV and decreased with increasing Bi content. The DC conductivity analysis indicated that small polarons contributed to the conduction mechanism, consistent with the calculated activation energy. The temperature dependence of the frequency exponent *s* was consistent with the correlated barrier-hopping model with two kinds of hopping mechanism (localised and translational) occurring in the system.

## Conflicts of interest

The authors declare that they have no conflict of interest.

## Supplementary Material
